# Public health round-up

**DOI:** 10.2471/BLT.20.010720

**Published:** 2020-07-01

**New Ebola outbreak**

A World Health Organization (WHO) vaccination team delivering Ebola virus vaccine in the Democratic Republic of the Congo where a new outbreak of Ebola virus disease was declared in the west of the country on 1 June. As of 9 June, more than 600 people had been vaccinated by WHO working in collaboration with the health ministry and other partners.

**Figure:**
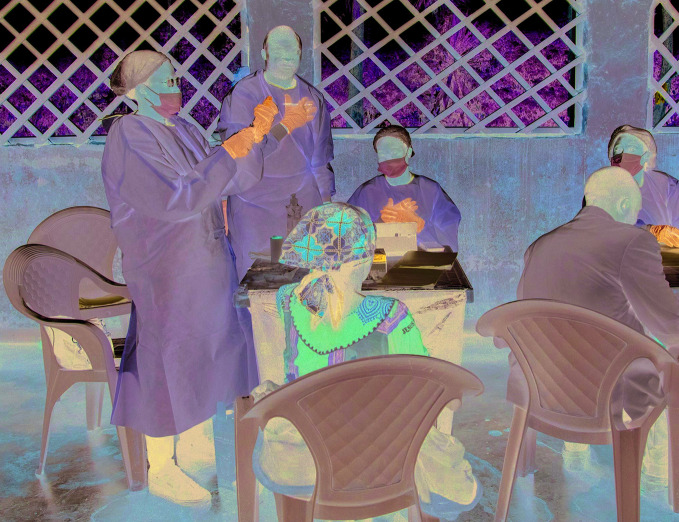
WHO/ Lindsay Mackenzie

## New Ebola outbreak

The Ebola virus now circulating in the west of the Democratic Republic of the Congo is genetically distinct from the virus that has infected more than 3400 people in the east.

The finding is the result of genetic sequence analysis done by the Democratic Republic of the Congo’s National Institute of Biomedical Research (INRB).

According to a 9 June report released by the World Health Organization (WHO) Regional Office for Africa, the INRB analysis also found that the virus in the latest outbreak, which was announced on 1 June 2020, is distinct from the virus that hit the same region in 2018.

The investigation is ongoing to determine the source of the new outbreak.

As of 9 June, WHO had more than 20 staff on the ground supporting the health ministry and partners responding to the outbreak in the city of Mbandaka and the rural community around the town of Bikoro. Additional staff were to arrive during the week, and vaccinators had been sent to the affected areas.

As of the same date, 12 people had been infected with the virus in the new outbreak, eight of whom died as a result.

As of 9 June, the outbreak in the east of the country had infected some 3463 people of whom 2280 had died.

https://bit.ly/3cMr4fp

## Pandemic response disrupts essential services

Services for the prevention and treatment of noncommunicable diseases (NCDs) have been severely disrupted in countries worldwide since the 2019 coronavirus disease (COVID-19) pandemic began.

A WHO survey completed by 159 countries during a 3-week period in May and released on 1 June revealed that in 150 of the countries, health workers had been partially or fully reassigned to support COVID-19 responses.

Services for hypertension care had been disrupted in 84 countries, while 78 countries reported disruptions in treatment of diabetes and diabetes-related complications. Sixty-six countries saw similar disruptions in cancer treatment and 49 countries in cardiovascular emergency services.

Rehabilitation services were also disrupted in 99 of the countries that responded to the survey, even though rehabilitation is often needed after the acute phase of COVID-19.

“The results of this survey confirm what we have been hearing from countries for a number of weeks now,” said WHO Director-General, Tedros Adhanom Ghebreyesus. “It’s vital that countries find innovative ways to ensure that essential services for NCDs continue, even as they fight COVID-19.”

https://bit.ly/3dQ4KmE

## Immunization services interrupted

Routine childhood immunization services have been significantly disrupted worldwide since March 2020, partly as a result of resources being directed towards COVID-19 responses.

According to a joint statement by WHO, the United Nations Children’s Fund (UNICEF), Gavi, the Vaccine Alliance, and the Sabin Vaccine Institute on 22 May, 68 of 129 countries reported moderate-to-severe disruptions, or a total suspension of vaccination services during March-April 2020. According to the statement, this is likely to affect around 80 million children under the age of 1 living in these countries.

https://bit.ly/3dXwHce

## WHO Foundation created

An independent grant-making entity that will support WHO’s efforts to address global health challenges was launched on 27 May.

Named the WHO Foundation, and based in Geneva, Switzerland, the entity, which is legally separate from WHO, will help broaden WHO’s donor base and work towards more sustainable and predictable funding, notably by allowing for contributions from the general public, individual major donors, corporations and other partners. The foundation’s board, which includes Thomas Zeltner, professor at the University of Bern, will assume all governance responsibilities.

The foundation will initially focus on emergencies and pandemic response, but it will also raise and disburse funds for all WHO global public health priorities in alignment with WHO’s 13^th^ General Programme of Work.

https://bit.ly/3cX01y1

## New funds for vaccines

World leaders pledged an additional US$ 8.8 billion for Gavi, the Vaccine Alliance, at the Global Vaccine Summit 2020, which took place on 4 June. The online summit included representatives from 52 countries, leaders from global health organizations, the private sector, vaccine manufacturers and civil society organizations.

The funding will support immunization of children in the world’s poorest countries against diseases such as measles, polio and diphtheria and will also be used to prepare health systems in anticipation of an eventual COVID-19 vaccine roll out.

According to Gavi, the new pledges will enable the alliance to vaccinate an additional 300 million children by 2025.

https://bit.ly/2BPcCGN

**Cover Photo**

Francine is an Ebola survivor who now looks after children while their parents recover in an Ebola treatment center in the Democratic Republic of the Congo. Dozens of survivors like Francine are able to look after the children because they are immune to the virus.

**Figure:**
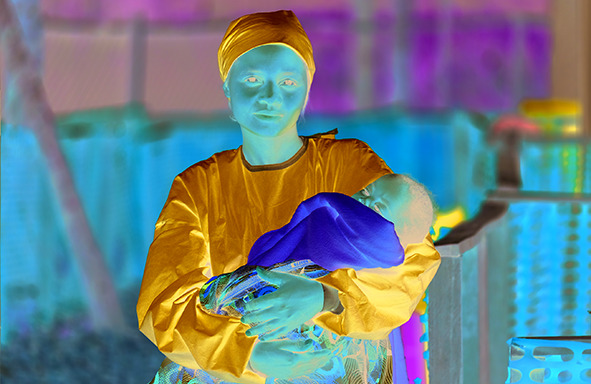
UNICEF/Patrick Rose

## Tobacco package ruling

The World Trade Organization’s (WTO) Appellate Body dismissed appeals from the Dominican Republic and Honduras against a WTO ruling regarding Australia’s tobacco plain packaging law.

The 10 June ruling puts an end to legal disputes, which began in 2012 and saw Cuba, the Dominican Republic, Honduras and Indonesia file legal complaints at the WTO regarding a law designed to help reduce demand for tobacco products.

The legal complaints argued that the Australian law restricted trade more than necessary to protect human health, and that it unjustifiably interfered with the use of tobacco company trademarks on packaging.

The WTO Appellate Body upheld an earlier WTO ruling that plain packaging is “apt to, and does, contribute” to Australia’s objective of improving public health by reducing tobacco consumption and exposure to tobacco smoke, is no more trade-restrictive than necessary for achieving that public health objective, and does not “unjustifiably encumber by special requirements” the use of trademarks in the course of trade.

WHO and the WHO Framework Convention on Tobacco Control (WHO FCTC) Secretariat provided the WTO panel with a brief on global tobacco control, summarized the public health evidence underlying tobacco plain packaging and the relevant provisions of the WHO FCTC.

https://bit.ly/2UIaYgE

## Harmful marketing of breast-milk substitutes

Countries are still falling short in protecting parents from misleading information about breast-milk substitutes, despite efforts to stop their harmful promotion – this is according to a new report by WHO, UNICEF, and the International Baby Food Action Network (IBFAN), which was published on 27 May.

Despite some progress in the prohibition of breast-milk substitute marketing, only 79 of the 194 countries analysed prohibit the promotion of breast-milk substitutes in health facilities and only 51 have provisions that prohibit the distribution of free or low-cost supplies within the health-care system. In addition, 19 countries prohibit the sponsorship of scientific and health professional association meetings by manufacturers of breast-milk substitutes.

“The aggressive marketing of breast-milk substitutes, especially through health professionals that parents trust for nutrition and health advice, is a major barrier to improving newborn and child health worldwide,” said Francesco Branca, Director of WHO’s Department of Nutrition and Food Safety.

https://bit.ly/30s2QEY

## Antibiotic research and development guidance

WHO released new target product profiles to provide detailed guidance for the research and development community working on the development of new antibiotics.

Released on 16 May, the profiles cover products needed to address enteric fever, gonorrhoea, neonatal sepsis and urinary tract infections.

Target product profiles outline the desired characteristics of products aimed at a particular disease or diseases, stating intended use, target populations and other desired attributes of products, including safety and efficacy-related characteristics.

https://bit.ly/2AnOn21

## Aid flown to Syria

WHO dispatched more than 80 tons of emergency medical supplies to support the health system in northeast Syria.

The 3-cargo consignment was airlifted from Erbil in the Kurdistan region of Iraq to Damascus, Syria between 10 to 12 June and included medicines, medical supplies and consumables.

The consignment is part of the humanitarian response to the Syrian crisis funded by the European Commission's Department for Humanitarian Aid and Civil Protection.

https://bit.ly/2N1E8Du

**Looking ahead**

July 11 - World Population Day. Virtual events related to reproductive health and gender equality.

July 22 - World Brain Day. Virtual events, including a webinar, to raise awareness of Parkinson’s disease.

July 28 - World Hepatitis Day. Virtual events to raise awareness of the global burden of viral hepatitis.

